# Multiple channels with interconnected pores in a bioceramic scaffold promote bone tissue formation

**DOI:** 10.1038/s41598-021-00024-z

**Published:** 2021-10-14

**Authors:** Xuesong Wang, Ziyan Nie, Jia Chang, Michael L. Lu, Yunqing Kang

**Affiliations:** 1 Department of Ocean and Mechanical Engineering, College of Engineering and Computer Science, Florida Atlantic University, Boca Raton, FL 33431 USA; 2 Department of Periodontology, University of Florida College of Dentistry, Gainesville, FL 32610 USA; 3 Department of Biomedical Science, College of Medicine, Florida Atlantic University, Boca Raton, FL 33431 USA; 4 Department of Biological Science, Faculty of Integrative Biology Program, College of Science, Florida Atlantic University, Boca Raton, FL 33431 USA

**Keywords:** Biotechnology, Biomaterials, Biomedical materials

## Abstract

Insufficient nutrition exchange and limited transportation of blood supply in a porous only scaffold often hinder bone formation, even though the porous scaffold is loaded with cells or growth factors. To overcome these issues, we developed a cell- and growth factor-free approach to induce bone formation in a critical-size bone defect by using an interconnected porous beta-tricalcium phosphate (β-TCP) scaffold with multiple channels. In vitro cell experimental results showed that multiple channels significantly promoted cell attachment and proliferation of human bone marrow mesenchymal stem cells, stimulated their alkaline phosphatase activity, and up-regulated the osteogenic gene expression. Multiple channels also considerably stimulated the expression of various mechanosensing markers of the cells, such as focal adhesion kinase, filamentous actin, and Yes-associated protein-1 at both static and dynamic culturing conditions. The in vivo bone defect implantation results demonstrated more bone formation inside multiple-channeled scaffolds compared to non-channeled scaffolds. Multiple channels prominently accelerated collagen type I, bone sialoprotein and osteocalcin protein expression. Fluorochrome images and angiogenic marker CD31 staining exhibited more mineral deposition and longer vasculature structures in multiple-channeled scaffolds, compared to non-channeled scaffolds. All the findings suggested that the creation of interconnected multiple channels in the porous β-TCP scaffold is a very promising approach to promote bone tissue regeneration.

## Introduction

The management of craniofacial bone defects caused by trauma, excision of pathological tissues or bone tumors remains challenging in the clinic. Currently, the major approaches to bone repair still use autologous bone grafts^1,2^. Autografts, such as iliac crest, scapula, and ribs are considered as a gold standard due to their distinguished osteoconductivity^3^. However, donor site morbidity and limited autologous tissue supply restrain their desirability^4^. Allografts and xenografts as alternatives to autografts, are less preferred, due to the risks of potential disease transmission and host immune rejections^5,6^. Therefore, artificial porous bone grafts, especially bioceramic scaffolds, which share similar chemical components and structures to natural bone have been extensively studied and brought many promising potentials in the repair of craniofacial bone defects^7^. Beta-tricalcium phosphate (β-TCP) scaffold is one of the most promising bioceramic porous scaffolds for bone tissue regeneration due to its outstanding mechanical property and osteoconductivity^8–10^. However, limited nutrient diffusion and transportation within the porous scaffold results in insufficient vascularization, low cell proliferation and survival rate, which in turn decreases bone formation and engraftment rate. These issues also shadowed porous β-TCP scaffold’ ability in bone regeneration, and in repair of large craniofacial bone defects^11–15^.

In our previous study, we found that multiple channels in a porous β-TCP scaffold promoted mandibular bone and vasculature formation in beagle dogs with the assistance of bone forming peptide-1 (BFP-1)^16^. In our recent study, we further demonstrated that without including any growth factors, multiple channels in a porous β-TCP scaffold significantly promoted endothelial cell attachment, infiltration, and angiogenesis exclusively in vitro, compared to non-channeled and single channeled β-TCP scaffolds^17^. The mechanistic study revealed that multiple channels induced a homogenous distributed shear stress field among the scaffold due to the interconnected channel-pore geometry, which activated certain integrins to promote endothelial cell infiltration and migration^17^. Nevertheless, it is still unclear whether the interconnected multiple channels-pores architecture can induce osteogenic stem cell differentiation in vitro and promote vascularization and bone regeneration in vivo.

According to the studies which displayed that shear stress plays an essential role in modulating mesenchymal stem cell osteogenic differentiation, collagen secretion, and matrix mineralization^18–21^, in this study we hypothesize that the multiple channels created inside porous β-TCP scaffold can activate shear force-driven mechanotransduction of cells, which can effectively stimulate the attachment, proliferation, and differentiation of mesenchymal stem cells in vitro, and sustainably activate osteogenesis and bone mineralization mechanism in vivo. To test the hypothesis, we utilized our own-developed bioreactor culture system to mimic the in vivo dynamic body fluidic condition. Traditional static culture was utilized as a control. Cell proliferation and differentiation studies on human bone marrow derived mesenchymal stem cells (hBMSCs) were carried out and specific osteogenic protein and genes were investigated in vitro. The underlying mechanisms by which interconnected channel-pore geometry regulating stem cells behaviors in vitro was explored through measuring various mechanosensing markers such as focal adhesion kinase (FAK), filamentous actin (F-actin), and Yes-associated protein-1 (YAP-1). To demonstrate the function of channels in bone regeneration and formation, an 8 mm critical-sized calvarial bone defect model in rat was used. Bone formation in vivo was histologically observed.

## Results

### Cell attachment efficiency and proliferation on the scaffolds

**Figure 1:**
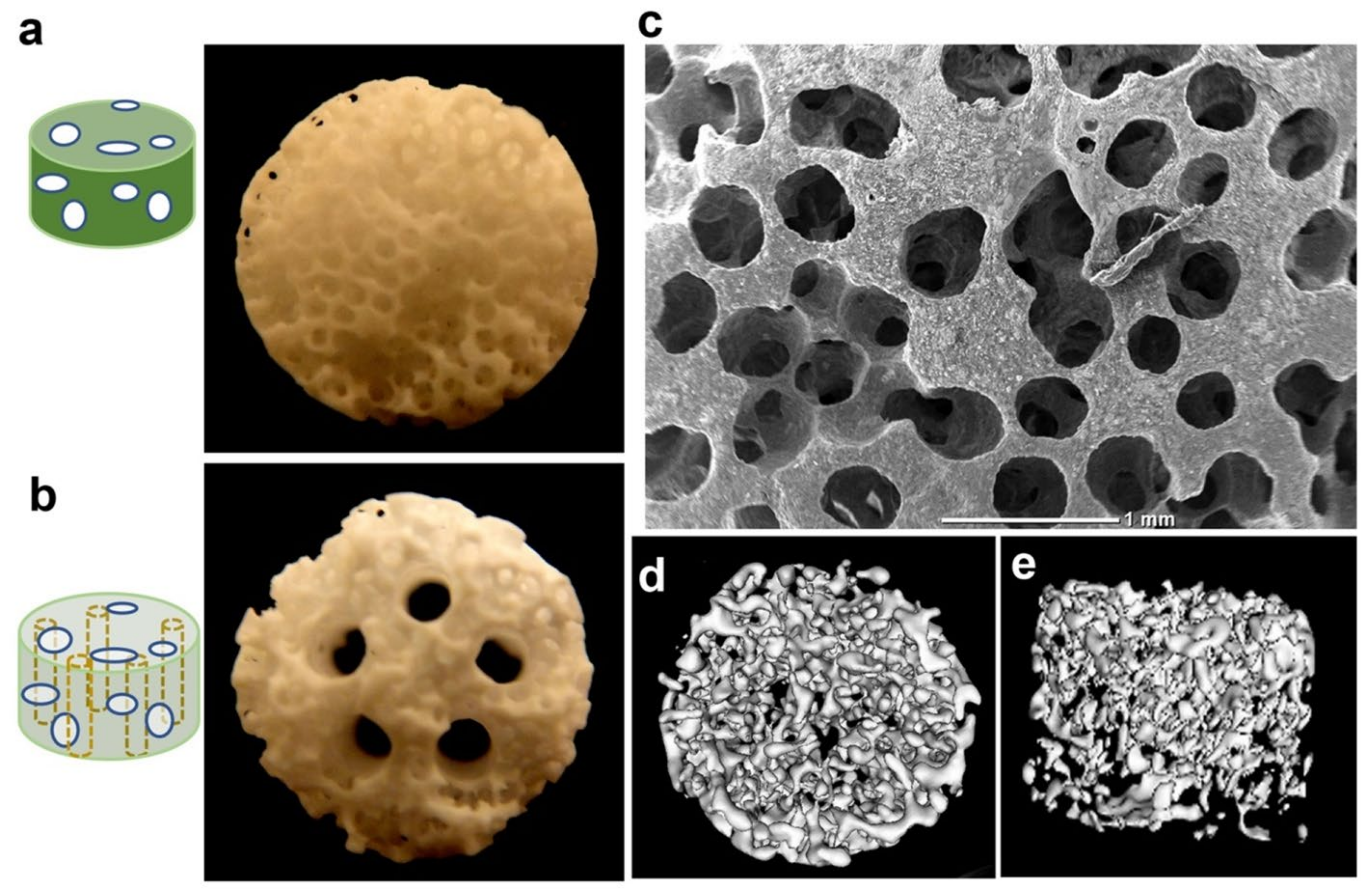
Images of scaffolds: two types of β-TCP scaffolds: (**a**) non-channeled porous scaffold; (**b**) porous scaffold with five straight channels. The diameter of the channel is 1 mm; (**c**) scanning electronic microscopic morphology of the non-channeled porous scaffold. Micro-CT images indicate the tortuosity of interconnected pores in the scaffold from top view (**d**) and side view (**e**).

Two geometrically different porous β-TCP scaffolds without channels and with five paralleled straight channels of 1 mm in diameter were fabricated in this study (Fig. 1a, b). The diameter of the scaffold is about 8 mm, and the height is around 5.5 mm. The pore size is around 550 μm. The interconnected porosity is approximately 80%^17^. From SEM images, we can see that the pores in the non-channeled scaffold are interconnected (Fig. 1c), and microCT images also showed the tortuosity of interconnected pores (Fig. 1d, e). After sintering, the porous scaffold was mainly composed of crystalline phased β-TCP according to the previous study^22^.

**Figure 2:**
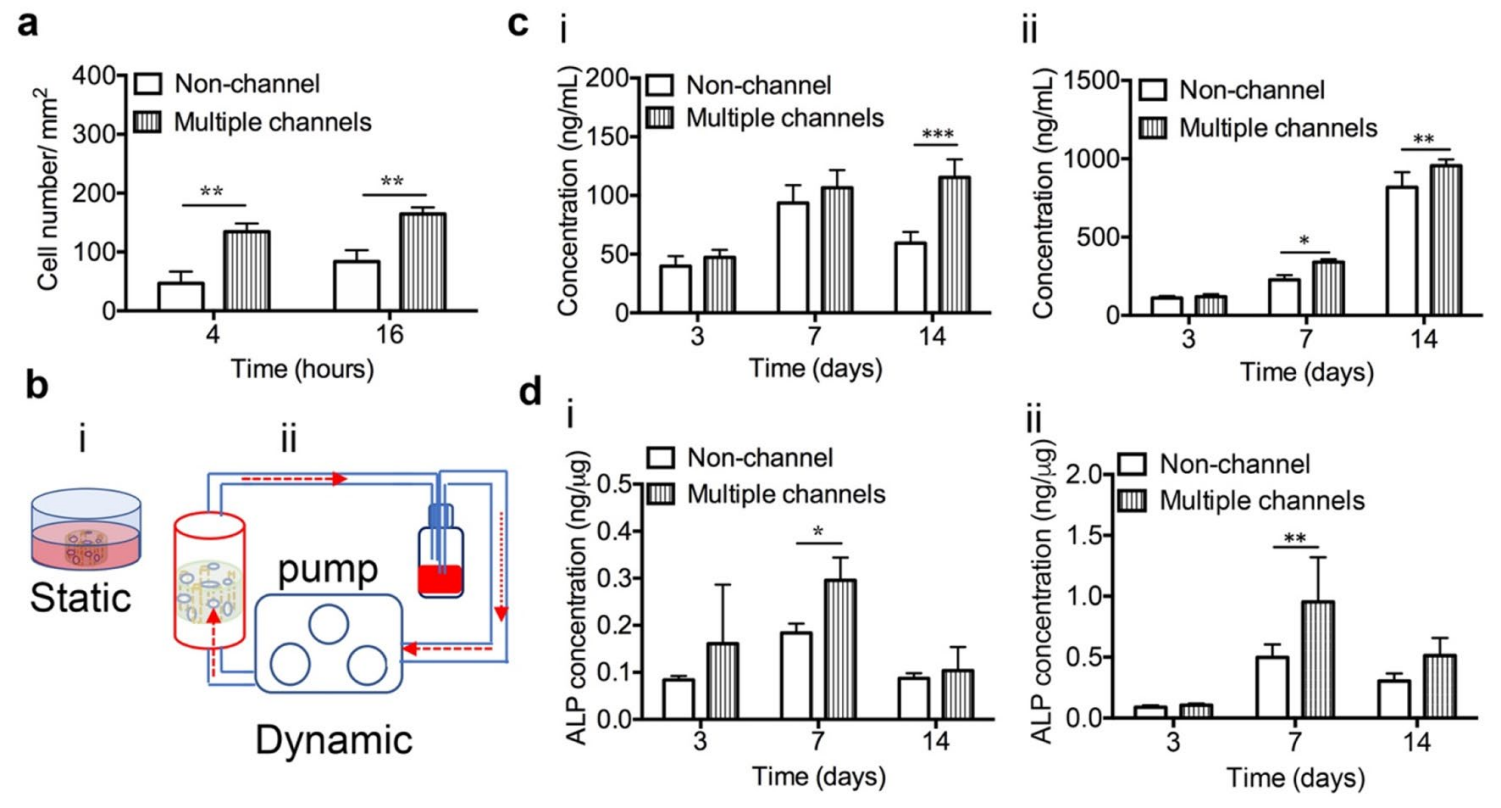
Cell attachment, proliferation, and ALP activity: (**a**) Cell number of hBMSCs attached on the two types of scaffolds after 4 and 16 h. (**b**) HBMSCs were cultured in the scaffolds under two culture conditions: (i) static culture; (ii) dynamic culture. (**c**) Cell proliferation of hBMSCs under the static condition (i) and dynamic condition (ii). (**d**) ALP concentrations of hBMSCs under the static condition (i) and dynamic condition (ii). (n = 3, *p < 0.05, **p < 0.01, ***p < 0.001).

hBMSCs were rotationally seeded onto the scaffolds, and the number of attached cells per unit area were measured respectively after 4 and 16 h. It showed that the number of attached cells was increased along with time from 4 to 16 h, and multiple-channeled scaffold significantly increased cell attachment, compared with non-channeled scaffolds (Fig. 2a).

To investigate the effect of multiple channels geometry on cell proliferation, we used dsDNA concentrations of hBMSC to indirectly index cell numbers. The dsDNA concentrations of total hBMSCs grown on both types of scaffolds under two culture conditions (Fig. 2b) were measured. Under the static condition (Fig. 2c, i), hBMSCs proliferated on both non-channeled and channeled scaffolds from 3 to 7 days. There is no significant difference in the dsDNA concentrations were detected between the two groups within 7 days. However, the dsDNA concentration of hBMSCs on non-channeled scaffold decreased at 14 days compared to it at 7 days, which in contrast, slightly increased on the channeled scaffold (Fig. 2c, i). The channeled scaffold resulted in a considerable higher dsDNA concentration at 14 days. Nevertheless, this cell proliferation pattern was not applied to that in the dynamic culture condition. Under the dynamic condition (Fig. 2c, ii), hBMSCs on both types of scaffolds continuously proliferated during the experimental period. Remarkably, the cell proliferation was significantly stimulated by multiple-channeled scaffolds compared to non-channeled scaffolds (*p* < 0.05). From 7 to 14 days, this difference of cell proliferation between the two groups became even more pronounced (*p* < 0.01). From these results, it demonstrated that multiple channels can significantly stimulate cell proliferation within 14 days. More interestingly, the absolute values of the dsDNA concentration under dynamic condition (~ 1000 ng/mL at day 14) were all significantly higher than those under static condition (~ 100 ng/mL at 14), regardless of time and scaffold types, even though the initial cell number that we seeded on the scaffolds was consistently same. This interesting result implied that the dynamic culture itself can promote cell proliferation significantly. In addition to that, multiple channels can stimulate that even further.

### hBMSC differentiation

The level of alkaline phosphatase (ALP) was measured as one way to explore the effect of multiple channels on cell differentiation in vitro. Results showed that, regardless of the culturing conditions, the ALP concentration of hBMSCs on both types of porous scaffolds increased with time and peaked at 7 days, and then decreased afterwards (Fig. 2d). There is no significant difference between the two types of scaffolds at most time points except for 7 days, at which, the ALP activity was significantly stimulated by multiple channels. These results demonstrated that multiple channels promoted early-stage cell differentiation of hBMSCs.

**Figure 3:**
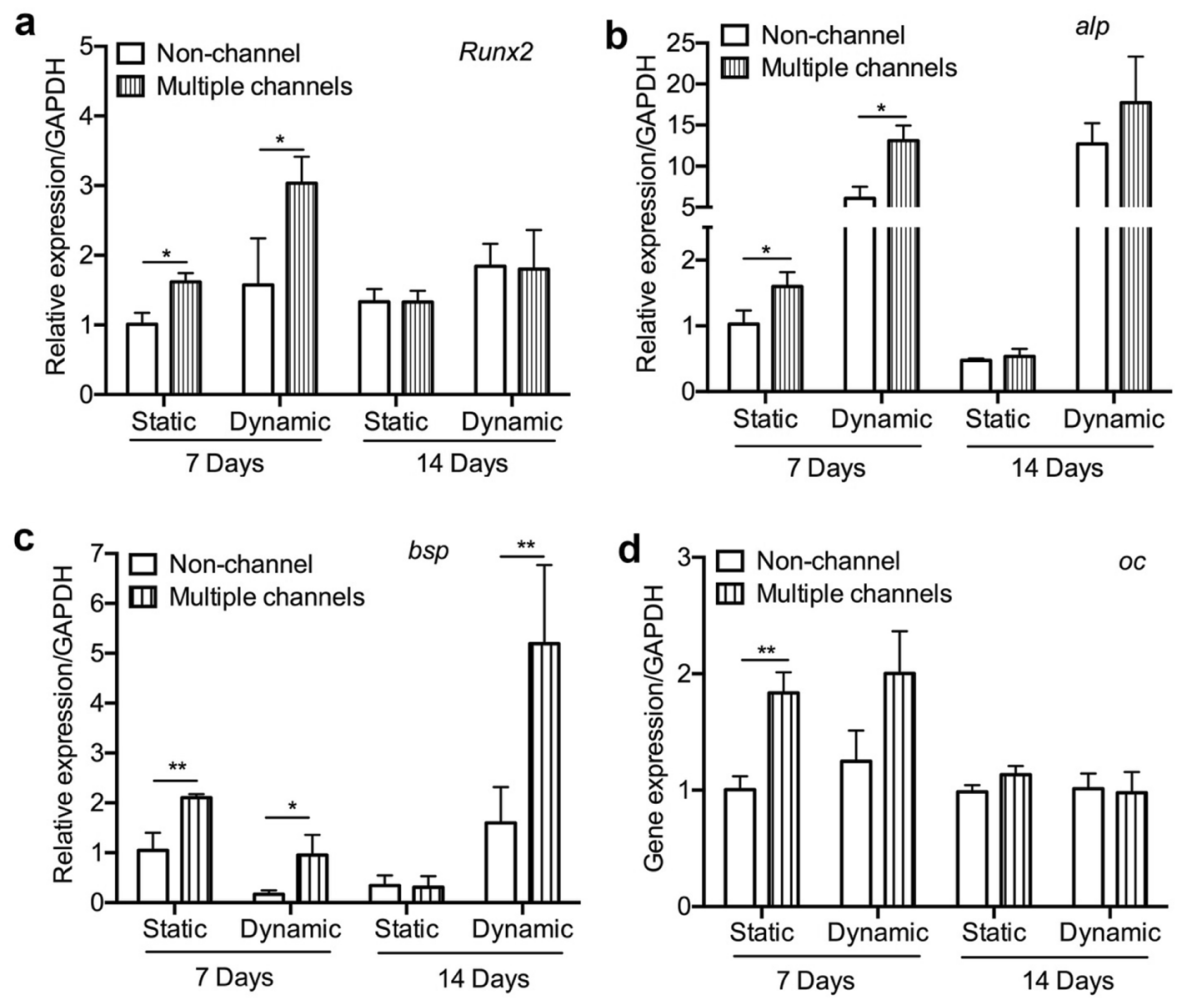
Osteogenic gene expression: osteogenic gene expression of hBMSCs on the β-TCP scaffolds under the static and dynamic conditions after 7 and 14 days. (n = 3, *p < 0.05, **p < 0.01).

Osteogenic-related gene expression was performed and measured by real-time PCR to further investigate the effect of multiple channels on hBMSC differentiation. Results indicate that multiple channels up-regulated the expression of *Runx2* gene by 1.6-fold under the static condition, and 2.1-fold under the dynamic condition after 7 days (Fig. 3a). However, after 14 days, no expression level variations were observed under both conditions. The expression level of *alp* was significantly promoted by multiple channels by 1.7-fold in the static condition, and 2.2-fold in the dynamic condition after 7 days (Fig. 3b). Both non-channeled and multiple channeled scaffolds significantly promoted *alp* expression under the dynamic condition compared to the static condition after 14 days. For *bsp*, the same trend as *alp* was observed at 7 days (Fig. 3c). At 14 days, under the static condition, no statistically significant difference was shown between the two groups (Fig. 3c). By contrast, under the dynamic condition, the multiple channels statistically enhanced *bsp* expression by 3.2-fold. The expression of *oc* was significantly promoted by multiple channels in the static condition at 7 days. At 14 days, *oc* expression remained almost the same level on both scaffolds in both culturing conditions, and there is no significant difference in the expression between the conditions and groups (Fig. 3d). In general, these results together indicated that multiple channels have the capacity to remarkably stimulate osteogenic-related genes expression of hBMSCs in vitro.

### Expression of mechanosensing markers: FAK, F-actin, and YAP-1

**Figure 4:**
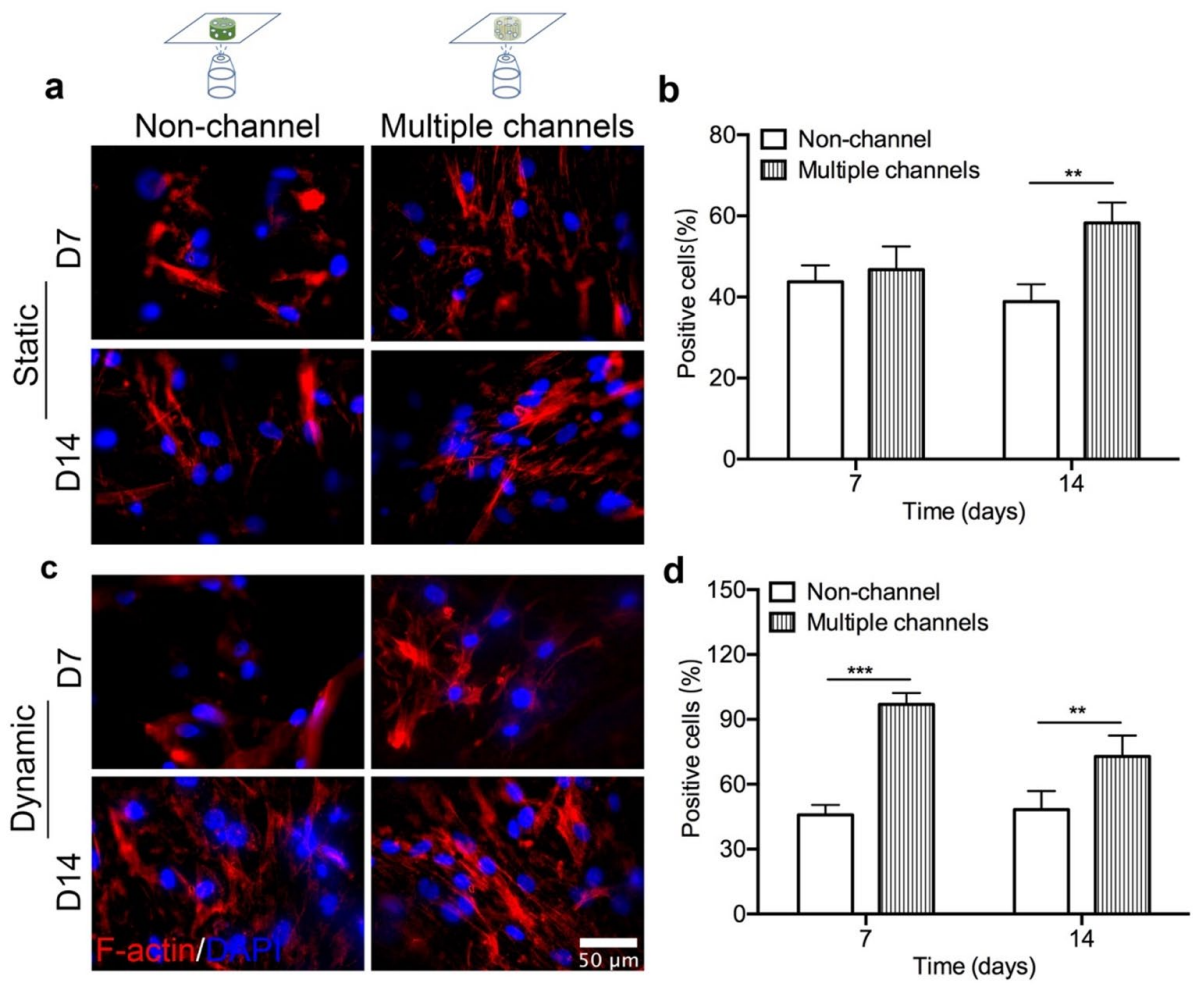
Mechanotransduction protein expression: stained scaffolds were placed on a glass cover slide for imaging. F-actin staining of hBMSC cells on the scaffolds under the static condition (**a**) and its F-actin expression percentage quantification (**b**), and under the dynamic condition (**c**) and its F-actin expression percentage quantification (**d**) at 7 and 14 days. F-actin expression percentage was significantly higher, and the positive signal was stronger in the multiple channeled scaffolds (n = 3, *p < 0.05, **p < 0.01, ***p < 0.001).

To investigate whether the interconnected channel-pore structure with the flow of dynamic fluid played a role in initiating the mechanotransduction pathways of hBMSCs, which promoted cell differentiation, the expression of various mechanosensing markers, FAK, F-actin, and YAP-1 were observed. F-actin staining of hBMSCs under the static condition shows that the actin fiber was not fully and strongly polymerized yet after 7 days, regardless of the scaffold geometries (Fig. 4a). However, the actin skeleton started to show highly polymerized and widely stretched after being cultured for another 7 days. The fibers are thinner and weaker on non-channeled scaffold, compared with that on multiple-channeled scaffold, which is thicker and stronger. The dynamic culturing condition remarkably intensified F-actin. More F-actin filaments started to widely spread on multiple-channeled scaffolds after 7 days. Whereas, compared to that, less F-actin on non-channeled scaffold were observed. After 14 days, F-actin filaments were highly strengthened and intensively bundled with each other on both types of scaffolds. Furthermore, multiple-channeled scaffold supported on strengthening fibrous bundle, forming more compact fibers inside cells. Semi-quantitative evaluation demonstrates that multiple channels significantly promoted the polymerization of F-actin after 14 days under the static culture condition (Fig. 4b). Dynamic condition accelerated the progress, and multiple channels started to stimulate F-actin polymerization (Fig. 4c). This significant stimulation of the intensity of F-actin fiber by multiple channels was continued from 7 days till 14 days in the dynamic condition (Fig. 4d).

**Figure 5:**
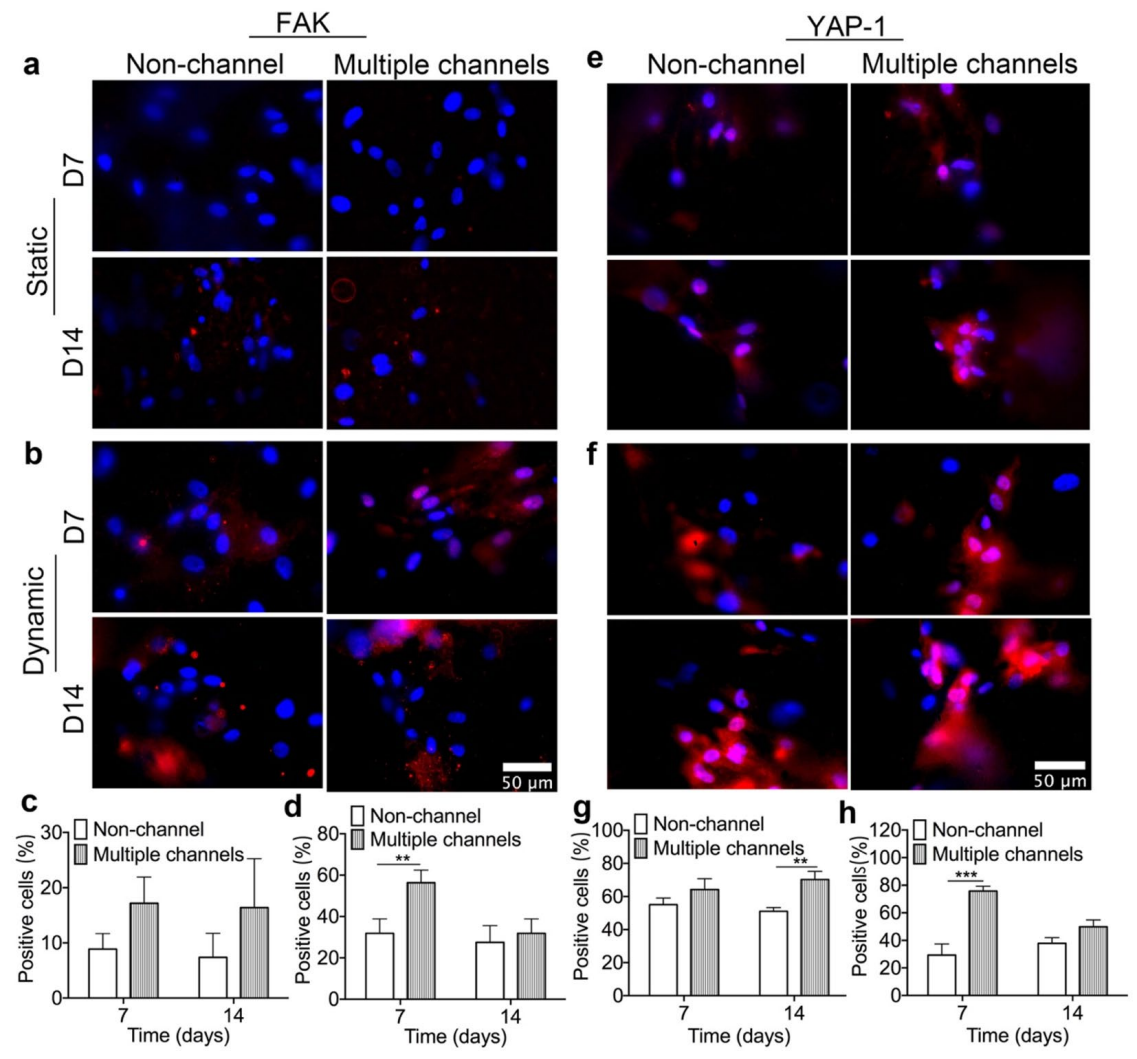
Mechanotransduction protein expression: immunofluorescent staining of FAK of hBMSC cells on the scaffolds under both static and dynamic conditions (**a**, **b**) and their FAK positive cells percentage quantifications (**c**, **d**); and Immunofluorescent staining of YAP-1 on hBMSC cells on the scaffolds under the static and dynamic conditions (**e**, **f**) and their YAP-1 positive cells percentage quantifications (**g**, **h**); (n = 3, *p < 0.05, **p < 0.01, ***p < 0.001).

For FAK, there are very few cells that expressed FAK on both types of scaffolds at 7 days, but there is relatively more FAK expression observed on the multiple-channeled scaffolds after 14 days under the static condition (Fig. 5a). However, interestingly, unlike static condition, more and stronger FAK signals were captured from the multiple-channeled scaffolds under the dynamic condition, whereas only a few cells positively expressed FAK on the non-channeled scaffolds after 7 days (Fig. 5b). After 14 days, there is no significant difference in the expression level of FAK on both types of scaffolds. Semi-quantification analysis shows that, under the static condition, though a slightly more FAK-positive cells exhibit at certain regions of multiple-channeled scaffolds, there is no statistically significant difference between two types of scaffolds on both two time points (Fig. 5c). Surprisingly, the combination of dynamic fluid and multiple channels together significantly enhanced the expression of FAK (*p* < 0.01) compared to non-channeled scaffold on 7 days (Fig. 5d). All these results imply that the multiple channels facilitate the dynamic fluid shear transmission inside scaffolds, which upregulates and activates the FAK expression.

YAP-1 was probed and there are only few cells positively expressing the protein on both types of scaffolds after 7 days under the static culture condition (Fig. 5e). They are weakly expressed and still mainly distributed in the cytoplasm area. However, after 14 days, multiple channels significantly stimulated YAP-1 expression in terms of the positively stained cell number, as well as the expressing strength and location inside the cells, which translocated from cytoplasm to nuclei, when compared with non-channeled scaffolds (Fig. 5e). Dynamic fluid accelerated this process and demonstrated the phenomenon earlier, in which condition, YAP-1 was strongly expressed in cell nuclei as well as cytoplasm area on multiple-channeled scaffold at 7 days, rather than on non-channeled scaffold (Fig. 5f). This significant difference disappeared after 14 days. The expressions of YAP-1 in cell nuclei and cytoplasm on channeled scaffold did not show significant increment. Semi-quantification shows that multiple channels slightly promoted YAP-1 expression after 14 days in the static condition (Fig. 5g). Under the dynamic condition, the expression was significantly stimulated by multiple channeled scaffolds after 7 days (Fig. 5h). All these results demonstrate that the geometry of multiple channels can significantly activate YAP-1 expression and translocation. The dynamic culturing condition could accelerate this mechanism.

### In vivo implantation and X-ray morphologies

**Figure 6:**
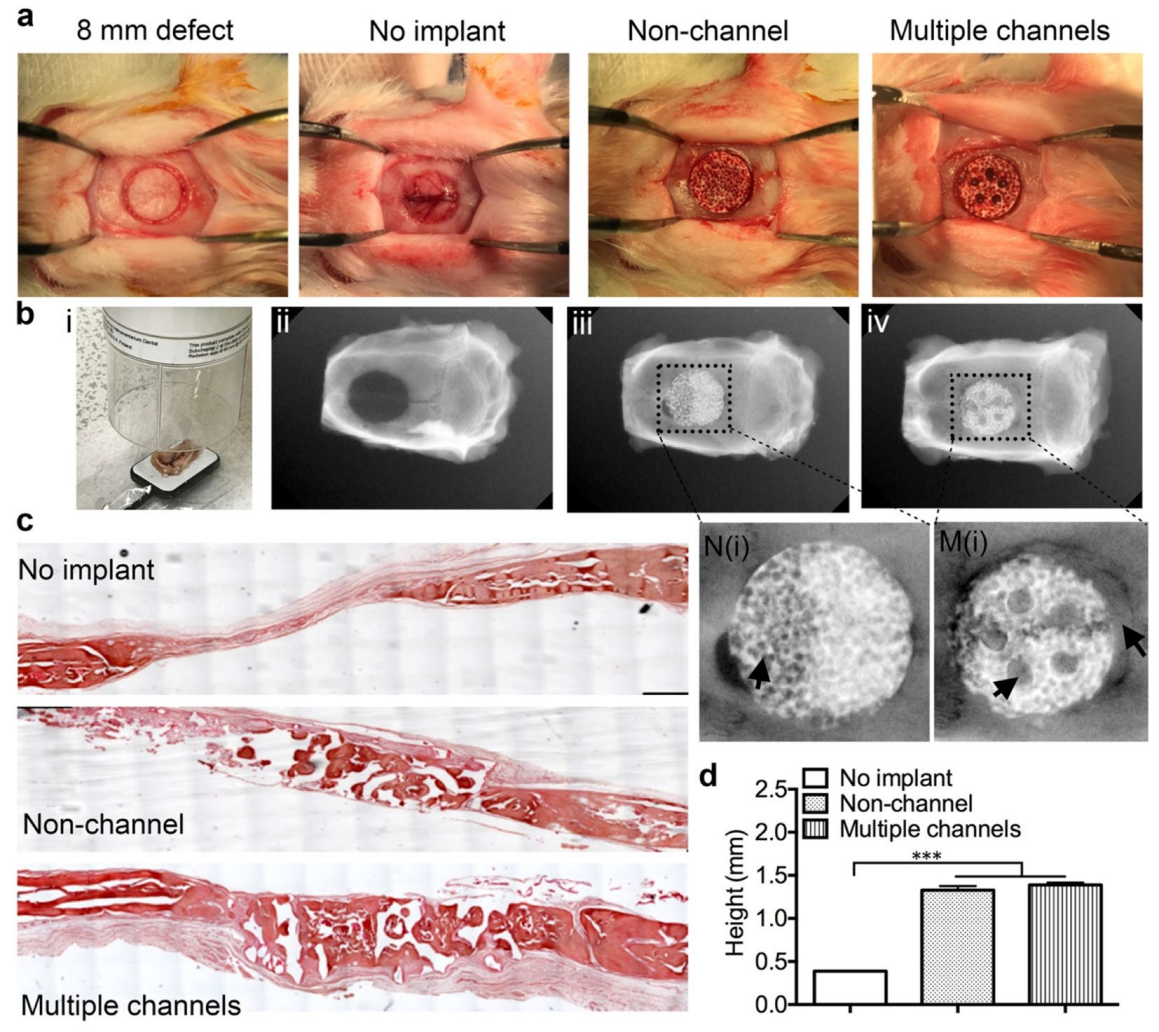
In vivo implantation and characterization: bone defects with 8 mm diameter and around 1.5 mm depth were created in the calvarial bone of rats (**a**), and scaffolds were implanted into the defects. X-ray microCT was scanning the explants (**b**, i) and X-ray photographical pictures on bone in the groups: (ii) no implant; (iii) non-channel; (iv) multiple channels. (**c**) H&E staining on the tissue sections of the three groups. (**d**) The height of the new formed tissue in the defect zone after 12 weeks. (n = 3, ***p < 0.001).

Scaffold implantation was carried out on a rat calvarial defect model (Fig. 6a) and characterized through X-ray to evaluate the function of multiple channels on bone regeneration and mineralization in vivo. After 3 months, the implanted scaffolds with surrounding native tissues was scanned by X-ray (Fig. 6b, i). Results showed that both types of β-TCP scaffolds promoted bone formation. The images revealed that there was no healing in the non-implantation group, and has a defective area remained with no new bone filled in (Fig. 6b, ii). In the non-channeled group, there are still some remained defective areas which were surrounded by the implant, as well as some pore area within the implant without any new bone tissue fillings (Fig. 6b, iii, N(i)). In the multiple-channeled group, it is showed that all the created bone defects, the channels area, and the pore areas were filled with new formed bone (Fig. 6b, iv, M(i)). Additionally, the radiopacity of regenerative bone is comparable to that of native calvarial bone in terms of the degree of mineralization (grey values of the image).

### Histological analysis

**Figure 7:**
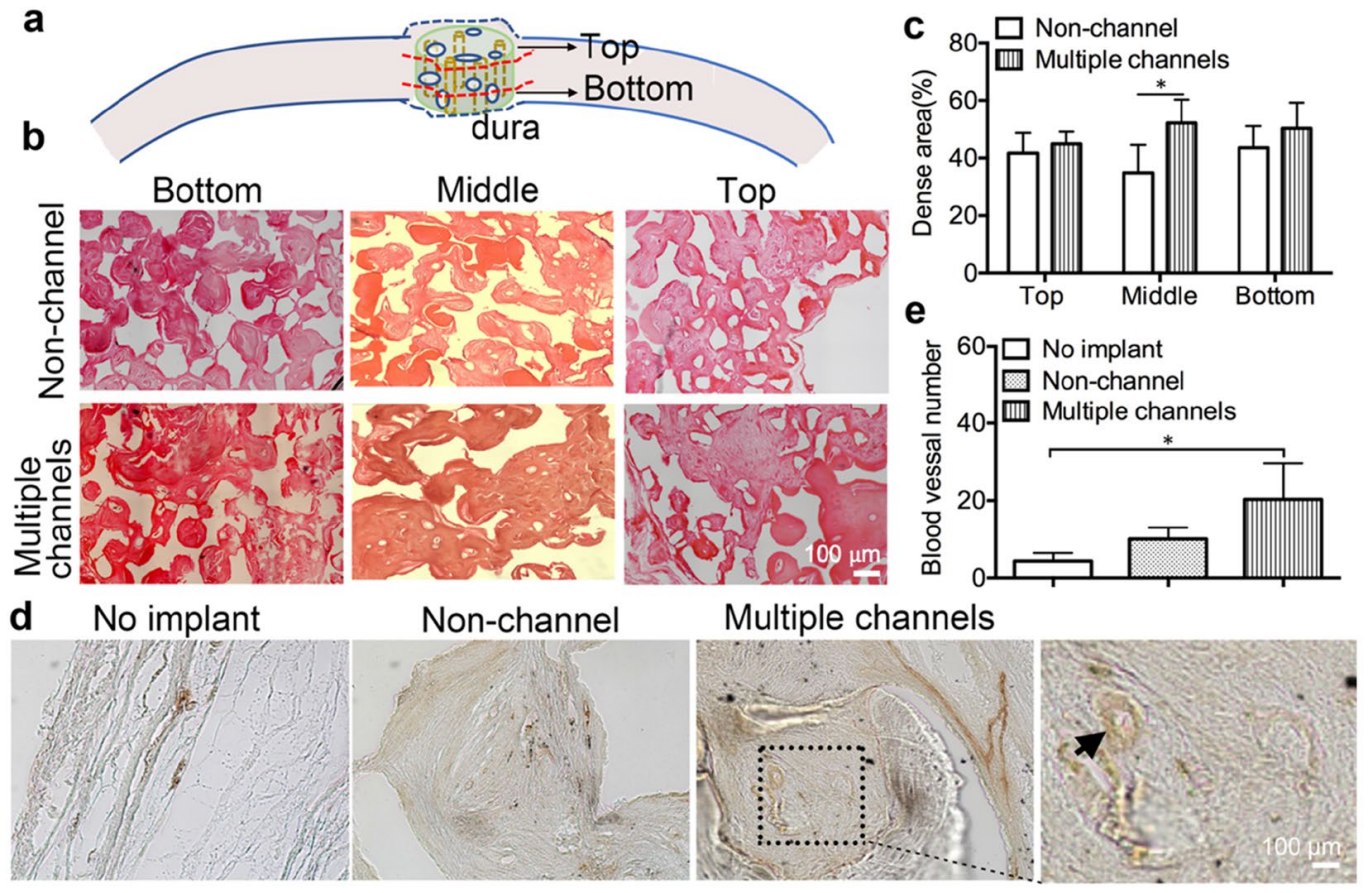
In vivo characterization: (**a**) schematic graph shows the three layers for cut sections of the implants when the embedded scaffolds were cut on a microtome. (**b**) H&E staining shows the structure of newly formed bone in the three layers of the two types of scaffolds. (**c**) Quantitative results of the dense area of new bone in the three different layers of the scaffolds. (**d**) Immunohistochemical staining of CD31 on the explants. (**e**) Quantitative results of the blood vessel number in the three groups based on the immunohistochemical images. (n = 3, *p < 0.05).

Lateral section of the central part of the implant/defect samples was stained by H&E staining. Results showed that there is a thin layer of 0.3 mm of soft tissue formed in the bone defect area, without any compact bone observed in the non-implant group. However, new bone tissue formed throughout the other two types of implants (Fig. 6c). After measuring the height of the new formed tissue in the defect zone, we found that the height of the bone defect (1.5 mm) was maintained by the implanted scaffolds after 12 weeks (Fig. 6d). We further performed H&E staining on the transversal sections of the proximal, middle, and distal segments from the dura side to the top of the implanted scaffolds (Fig. 7a). We found that there is extensive bone growth in all the layers of both non-channel and multiple-channeled scaffolds, but the density of the formed bone is different. In the bottom layer, the density and dense area of the formed bone tissue are similar in the two groups (Fig. 7b). However, with the location of the layers became further away from the dura layer, the formed bone tissue in the two groups became different. There are some small islands of fibrous bone tissue observed in the middle segments of the non-channeled scaffold, but larger areas of compact bone were formed inside multiple-channeled scaffolds (Fig. 7b). The semi-quantitative analysis shows that the ratio of compact bone to total tissue in the middle layer of the channeled scaffolds is much higher than that in the non-channeled scaffolds (Fig. 7c). In the top layer, the density and dense area ratios in the two group became similar again. These results imply that new tissues grew easily from two ends (bottom layer and top layer, where tissues contacted the two ends of the scaffolds) into the scaffold. However, the new bone tissue may not easily reach to the middle part of the non-channeled scaffold, but the channels facilitated the ingrowth of new bone tissue to the middle part even the whole area of the channeled scaffold. These H&E staining results illustrated that multiple channels in porous β-TCP scaffold stimulated new bone formation and maturation. The formation of stimulated new bone tissue may be related to the effective vascularization in the channeled scaffold. To prove this point, we used immunohistochemical staining of angiogenic marker CD31 to observe the formation of blood vessels.

Angiogenic marker CD31 staining showed that only small tubular-like blood vessels were observed on both non-channeled scaffold and no-implant group, but there was longer and tissue penetrative vasculature structure formed in the multiple-channeled scaffolds (Fig. 7d). We also observed that there are erythrocytes in the lumen of blood vessels (magnified image). Although there is no statistically significant difference in vasculature number between multiple-channeled scaffolds and non-channeled scaffolds (Fig. 7e), there is still significant difference in terms of the size of the formed blood vessels. This result implied that multiple channels have the potential of enhancing blood vessels formation, thus inducing more bone tissue formation in the channeled scaffolds.

### Immunohistochemical staining

**Figure 8:**
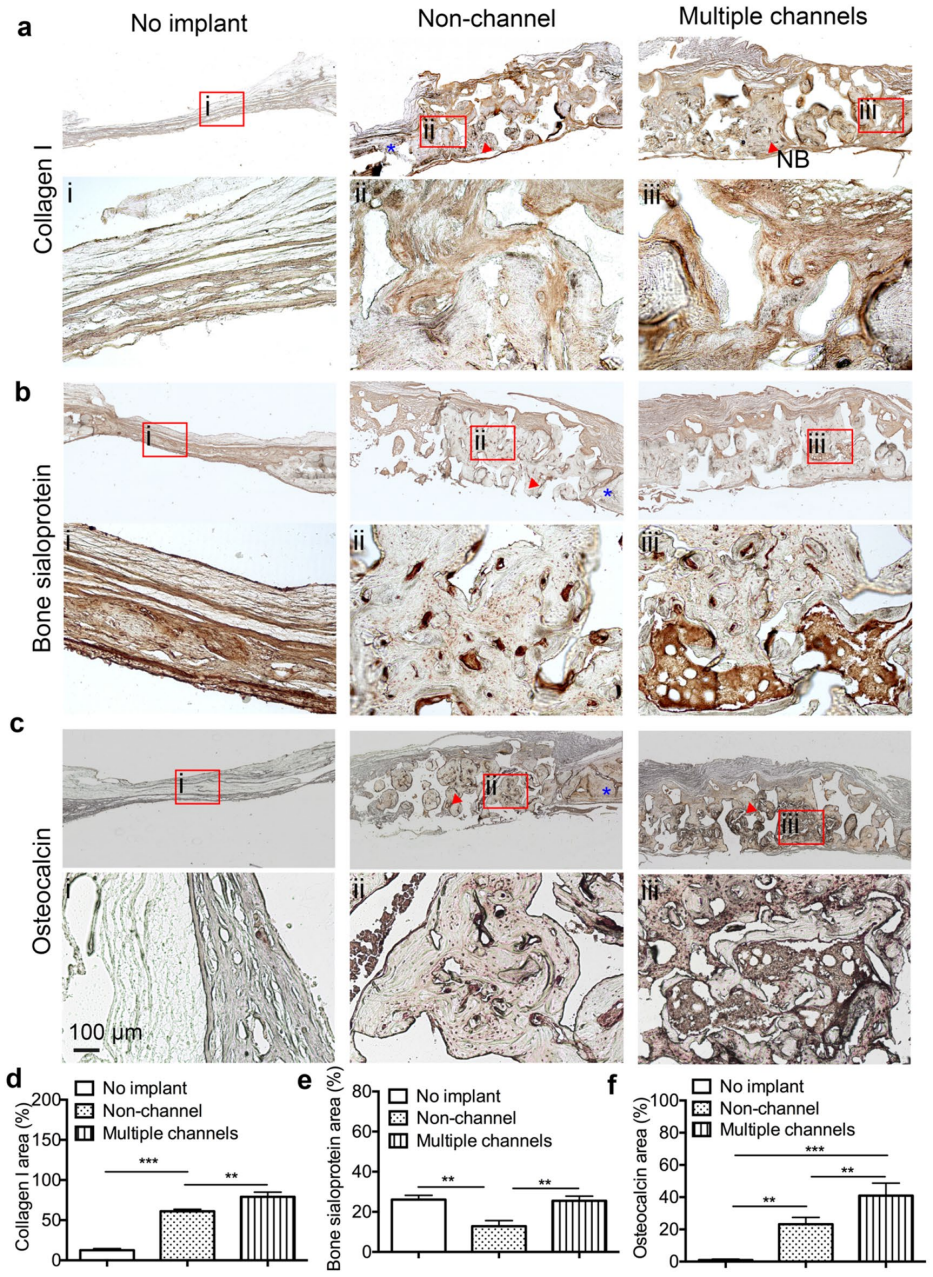
Immunohistochemical staining: the immunohistochemical staining of collagen type I (**a**), bone sialoprotein (**b**), and osteocalcin (**c**), and their respective quantitative results from the images of collagen type I (**d**), bone sialoprotein (**e**), and osteocalcin (**f**). Magnified images from a local spot of the three groups: (i) no implant, (ii) non-channel, and (iii) multiple channels. Red arrows mean new formed bone tissue matrix, and blue * means native bone matrix. These result show that channeled scaffolds significantly promoted the formation of more bone matrix than non-channeled scaffolds and no-implant groups. (n = 3, *p < 0.05, **p < 0.01, ***p < 0.001).

A series of immunohistochemical straining of bone matrix proteins including collagen type I, bone sialoprotein (BSP), and osteocalcin (OC) were carried out. The results showed that collagen type I was expressed in all three groups (Fig. 8a). The patterns of stained collagen type I in both non-channeled and multiple-channeled scaffolds are similar to that in native calvarial bone (Fig. 8a, ii, iii), compared to the no-implant group (Fig. 8a, i). There are few collagen fibers observed on the distal layer (from dura side) in the non-channeled scaffold group (Fig. 8a, ii), but remarkable collagen fibers were homogenously distributed around the whole multiple-channeled scaffolds (Fig. 8a, iii). Semi-quantification of the positively stained collagen type I from these images displays that the ratio of total collagen type I to the whole tissue area in the multiple-channeled scaffolds is significantly higher than that in the non-channeled scaffolds (Fig. 8d). Besides that, this ratio in both two type of scaffolds is considerably higher than that in no implant group. For BSP, we found that it is highly and uniformly presented in the no-implant samples, but not in the non-channeled scaffolds (Fig. 8b, i). In the non-channeled group, BSP is only localized along the peripheral areas and at the center of mineralized bone tissue (Fig. 8b, ii). However, in the multiple-channeled scaffolds, it is not only localized at the same regions as non-channeled scaffolds, but also widely spread at the core regions of the channel’s area (Fig. 8b, iii). Semi-quantitative analysis shows that the expression ratio of BSP in the multiple-channeled scaffolds is significantly higher than that in the non-channeled scaffolds (Fig. 8e). Surprisingly, positive BSP staining is statistically higher in the no-implant group compared with the non-channeled group as well, although no newly mineralized bone was observed inside this group. We further stained a late stage mineralized matrix protein, OC. We found that there is no positively stained OC signal found in the no-implant group (Fig. 8c, i), but OC was spread and confined to the area where compact bone formed, or mineralizing tissues are adjacent to the compact bone in both non-channeled and multiple-channeled scaffolds (Fig. 8c, ii, iii). Furthermore, multiple-channeled scaffolds express significant higher OC than non-channeled scaffolds (Fig. 8c, iii). Similarly, semi-quantitative analysis shows that the expression ratio of OC in the multiple-channeled scaffolds is significantly higher than that in the non-channeled scaffolds (Fig. 8f).

**Figure 9:**
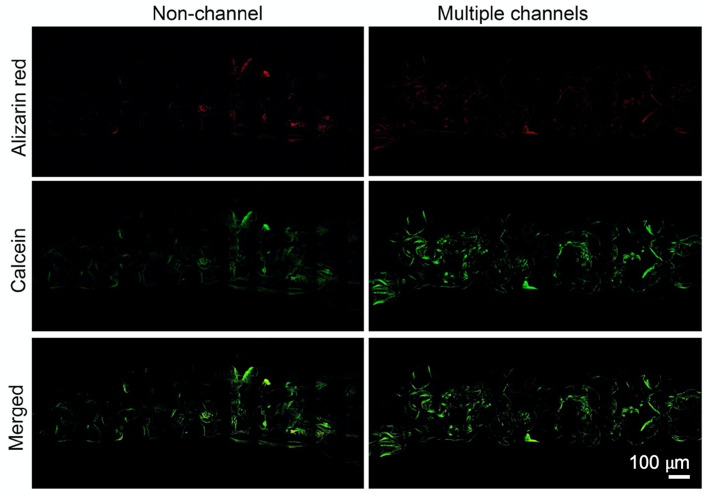
Flurochrome labeling: fluorescent labeling of new bone formation over time. Images in red and green present the calcified tissue, respectively.

Dynamic deposition of mineralized tissue in the non-channeled and multiple-channeled scaffolds is demonstrated through fluorescent images of the sections from fluorochrome labeled samples (Fig. 9). The results show that the amount of fluorescent alizarin red signal is much less than calcein green signal, and most of the alizarin red signal is overlapped and covered by calcein green signal. In the non-channeled scaffolds, the signals are only confined within half area of the whole structure. However, there are more and stronger mineral deposition signals widely dispersed around everywhere inside the multiple-channeled scaffolds, compared to the non-channeled scaffolds.

## Discussion

Using porous osteoconductive β-TCP bioceramic scaffolds for bone regeneration has been widely reported and studied^23,24^. However, challenges such as maintenance of sustainable nutrient diffusion that directly leads to an effective bone formation are still unsolved^25,26^. Insufficient vascularization and limited internal–external nutrient exchange delayed osteogenesis and bone regeneration^11–15^. Strategies on modifying the scaffolds with growth factors lost their superiority due to the challenges such as short half-life and unwanted physiological side effects^27,28^. In our previous studies we’ve already proved that interconnected channels-pore geometry generated a relatively strong and homogeneously distributed fluid shear stress field inside the scaffold, which contributed to endothelial cell migration and promoted angiogenesis in vitro^17^. Further, in this study, we continued to utilize the discovered mechanotaxis property of multiple channels to induce bone mineralization and formation in vivo. We verified that the interconnected channels-porous architecture significantly promoted hBMSCs proliferation and differentiation in vitro through activating mechanotransduction pathway, and considerably stimulated osteogenesis, mineralization, and bone formation in vivo, compared to the non-channeled scaffolds.

All these findings indicated that the architecture of multiple channels with interconnected pores play a significant role in cell proliferation, differentiation in vitro and bone tissue formation in vivo. These results also implied that without any additional biological components (cells, growth factors), the new architecture itself also activated an endogenous bone formation cascade. The channels facilitated the circulation of dynamic fluid and generated a homogenously higher fluid shear, which can be sensed by cells and in turn triggered the cells mechanotransduction signaling pathways that correlated with cell proliferation and tissue formation. This finding was consistent with the research of Kapur et al., in which they demonstrated that fluid shear stress triggered human osteogenic cell proliferation^29^. What’s more, it is paramount that an uninterrupted differentiation process of multipotent mesenchymal stem cells needs to be initiated and sustained along with osteogenesis and bone formation^30,31^, which is a condition that can be effectively maintained by the interconnected channel-pore structure of the scaffold. Our results suggested that multiple channels play an indispensable role on hBMSCs differentiation by promoting alkaline phosphatase activity and up-regulating relative osteogenic gene expressions. The expression of the four genes investigated in this study follows a chronological order. According to that, interestingly, we found the dynamic fluid not only promoted the expression level of the genes, but also prolonged the expression time of each gene. This dynamic fluid condition synergizing with multiple channels makes the gene expression pattern become more pronounced. All these observations on cell differentiation demonstrate that the interconnected channels-pores structure kept cell differentiation process sustainably in vitro. These findings are consistent with other people’s studies. Bjerre et al. reported that the expression level of *Runx2* on scaffolds that were cultured in a perfused condition was much higher than that in the static culturing condition at very initial stage of cell differentiation^32^. Yourek et al. and Kreke et al. reported that fluid-flow-induced shear stress significantly promoted ALP activity and up-regulated *bsp* and *oc* gene expression^33,34^.

It’s been also reported that fluid shear stress could activate Hippo/YAP pathway, the inducer of MSCs differentiation, through transferring the signal from FAK to YAP, which stimulated the production of Runx-2 and promoted osteogenesis^35^. In this study, it aroused our interest to see, without any biological components involved, whether mechanotransduction pathway of osteogenesis was activated by multiple channels, and acted as the underlying mechanism of enhancement of cell differentiation^36^. The immunofluorescent staining displays that multiple channels facilitated the diffusion of dynamic fluid, which activated the expression of FAK. FAK assisted the transmission of fluid shear stress signal and promoted the polymerization and formation of F-actin fibers, which upregulated as well as triggered the translocation of YAP-1 (Figs. 4, 5). The remarkable activation of proteins which are highly affiliated with osteogenic mechanotransduction pathway in multiple channeled scaffold, indicated that a relatively higher fluid shear force was initiated inside the scaffold, thus significantly promoting the expression of osteogenic genes and cell differentiation in vitro.

In vivo results further demonstrated that multiple-channeled scaffold promoted osteogenic collagen type I, BSP, and OC protein expression and sustainably enhanced the formation of dense bone. These results imply that during intramembranous bone formation, collagen fibers acted as a woven matrix for incorporating non-collagenous proteins such as BSP to initiate mineralization and modulate calcium phosphate mineral deposition^37–39^. Thus, a sustainable collagen production is essential for fully bone regeneration. In Roah’s study, they found that the early osteogenic markers were not highly presented in heavily calcified bone^37^. In our study, we also found that more areas on multiple-channeled scaffold were filled with highly mineralized matrix, which has the appearance very similar to native calvarial bone. Collagen type I is homogenously distributed everywhere in the channeled scaffold, rather than that only distal layer from dura side is highly positively stained in the non-channeled scaffold. However, collagen is only observed on the above half of the non-channeled scaffold. BSP, one of the most abundant bone non-collagenous proteins, has collagen-binding sequence and negatively charged^40^. During the bone regeneration process, BSP binds to collagen fibers and then initiates the deposition of the calcium phosphate crystals to the site^41,42^. Therefore, BSP is essential on bridging upstream and downstream osteogenesis and bone mineralization. In our study we found that BSP protein expressed significantly higher in the multiple-channeled scaffolds than non-channeled scaffold (Fig. 8b). In addition to that, we observed that BSP distributed widely at the areas where un-matured fibrous tissue is located, such as the center of channel area, and no-implant samples. This observation is quite similar to that of collagen type I, where more staining was seen at the non-matured tissue areas. This finding could be explained by the study of Roah, in which collagen I and BSP were found to disappear when tissue became highly mineralized and calcified to bone matrix^37^. All these discoveries indirectly indicated the reinforcement of rapid bone formation by multiple channeled scaffolds, which might sustainably activate self-circulative osteogenic mechanisms and promoted persistently bone formation. However, in the non-channeled scaffolds, the positive staining area of both collagen I and BSP is limited. This implies that bone mineralization and formation in the non-channeled scaffold are non-sustainable and delayed. Except for that, we found multiple channels significantly promoted the expression of late-stage bone mineralization marker osteocalcin, and considerably increased mineral deposition in the channeled scaffold through observing fluorochrome labeled samples. All these findings indicated that multiple channels prompted bone formation and maturation. The underlying mechanism may include but not limited to the surrounded cells and tissues in response to the mechanical shear force that was generated by the interconnected channels-pore architecture^17^. However, the detailed signaling pathways that correlate mechanosensation or mechanotransduction to osteogenesis and bone formation in vivo may need further studies to be verified. Even so, this new porous β-TCP scaffold with multiple channels provides a promising approach with cell- or growth factor-free component to induce bone tissue regeneration.

## Conclusion

In this study, we demonstrated that the creation of multiple channels in a porous β-TCP scaffold promoted hBMSCs differentiation and osteogenesis in vitro, and sustainably induced bone formation in vivo. The interconnect channels-pore geometry in porous β-TCP scaffolds had significant functionality to stimulate new bone formation. The findings implied that the architecture of multiple channels-pores in the scaffold act as a promising stimulator to promote bone regeneration.

## Materials and methods

### Preparation of β-TCP scaffolds with multiple channels

Two geometries of interconnected porous β-TCP scaffolds were fabricated by using a template-casting method that was reported in the previous study^22^. One is porous β-TCP scaffold, the other is porous β-TCP scaffold with five channels. The diameter of each channel is 1 mm. The five channels are homogenously distributed in the porous β-TCP scaffold. Briefly, β-TCP slurry was prepared by stirring mixture of β-TCP nano-powder (Nanocerox, Inc, Ann Arbor, Michigan), carboxymethyl cellulose powder, dispersant (Darvan C), and surfactant (Surfonals) with distilled water (Fisher Scientific). After filling the paraffin beads into the two types of customized modes, β-TCP slurry was casted into the molds and solidified in ethanol for two days, followed with a gradient ethanol dehydration. The completely dried β-TCP green-bodies were then sintered for 3 h at 1250 °C. The fully sintered scaffolds were then used in the in vitro and in vivo studies.

### Characterizations

The pore morphologies of the porous scaffold were observed by scanning electron microscopy (SEM). The dried scaffold was sputter-coated with gold for observation under a benchtop SEM (JEOL, JCM-6000Plus). The tortuosity of pores was observed by a MicroCT scanner (Imtek Micro CAT II, Knoxville, TN) at a resolution of 80 μm.

### In vitro cell attachment quantification

To measure whether hBMSCs’ attachment efficiency was promoted by channels, six scaffolds of each type were placed in the wells of 24 ultra-low adherent well plates (Corning, NY). One scaffold per well was set. hBMSCs from Lonza were cultured with MSCGM medium (Lonza, Basel Switzerland) under a standard cell culture condition. Two milliliters of hBMSCs suspension with a concentration of 5 × 10^5^ cells/mL were added into each well. To dynamically seed cells onto the scaffolds homogenously, the well-plates were then placed on a 3D platform rotator (Fisher scientific, 3D platform rotator, Hampton, NH) and rotated at 30 rpm in CO_2_ incubator at 37 °C. At 4 and 16 h, 3 wells with each type of scaffolds were used to determine the cell attachment efficiency according to our published method^17^. Briefly, each scaffold was taken out and washed with 1 × PBS three times. All washed PBS, the leftover media, and the trypsinized content from each well were collected into a 15 mL centrifuge tube. The cell number was counted from each tube. Final cell attachment efficiency was determined by measuring the cell number per scaffold surface area and followed the equation: *Na* = (*Ns* − *Nc*)/*As*, where *Na* stands for the number of attached cells per unit area, *Ns* stands for the initially seeded cell number, and *Nc* stands for the collected cell number in the tube, and *As* stands for the surface area of each type of scaffolds. The surface area of each scaffold was calculated through the dimensions and volumes of the scaffold and its pores.

### hBMSCs proliferation and osteogenic differentiation

Channels’ function on hBMSCs proliferation property was investigated in both static and dynamic culturing conditions. One hundred microliters of 10^5^ hBMSCs suspension was pre-seeded onto each scaffold and incubated for an hour at 37 °C, after which equal and enough MSCGM medium was added to each well to fully cover the scaffolds and kept culturing for another 24 h. The cells/scaffolds were kept culturing in 24-well plates with MSCGM medium for static condition measurement. A continuous 3-, 7-, and 14-days’ culture with medium changed every 2 days was followed. For dynamic condition measurement, after 24 h of initial culture, all the cells/scaffolds were transferred into a dynamic circulating bioreactor system where fresh MSCGM medium was circulated through the cells/scaffolds at a rate of 10 μL/min for 3, 7 and 14 days as well, according to the setting of the dynamic culture system we used in previous study^17^. At the end of each time point, samples from both static and dynamic culturing conditions were collected and rinsed with 1 × PBS twice and stored at -80 °C.

Fluorometric assay was utilized to measure the cell proliferation property quantitatively. Briefly, the cell lysate was collected by immersing all the stored cells/scaffolds samples with 0.2% Triton X-100 in 1 × TE buffer solution and followed by three freeze–thaw cycles. For each cycle, cells/scaffolds were frozen at − 80 °C for 20 min, and then thaw to 37 °C for another 20 min. After that, the content of dsDNA from each scaffold was measured by using a Quant-iT™ PicoGreen™ dsDNA Assay Kit (Invitrogen, Carlsbad, CA) according to the manufacturer’s instruction. All the samples were read through Spectra Max Gemini EM plate reader with 480/520 nm excitation/emission wavelengths^43^.

Alkaline phosphatase (ALP) activity was measured as one way to investigate the effect of channels on hBMSCs osteogenic differentiation. The cells/scaffolds were cultured in both static and dynamic systems as described above as well. However, after 24 h pre-seeding in MSCGM medium, the culturing media was changed to osteogenic differentiation media, which contained 10% FBS, 10 mM β-glycerophosphate, 10 nM dexamethasone, 50 mg/mL ascorbic acid, and 1% PSA, other than MSCBM. The cells were continuously cultured for 3, 7 and 14 days, and samples were rinsed with 1 × PBS, collected and stored in − 80 °C at each time points for further tests.

To determine the cell ALP activity quantitatively, the total protein was isolated through three times of 80 °C to 37 °C freeze–thaw cycles as well by immersing all the stored cells/scaffolds osteogenic differentiation samples within 0.2% Triton X-100 solutions. The total protein was measured through Pierce™ BCA Protein Assay Kit (Thermofisher Scientific), and read by Spectra Max 190 plate reader at the wavelength of 562 nm. The total ALP was determined through Quantitative Alkaline Phosphatase ES Characterization Kit (EDM Millipore, CA), and read by Spectra Max 190 plate reader at the wavelength of 405 nm. Final cell ALP activity was determined through the following equation: *C*_*Aa*_ = *Ca/Cp*, in which *C*_*Aa*_ stands for ALP activity, *C*_*a*_ stands for the total alkaline phosphatase amount and *C*_*p*_ stands for the amount of total protein of each sample^44^.

### Real time PCR

Osteogenic differentiation related genes were run by real time PCR. Human BMSCs were cultured with osteogenic differentiation media in the same static and dynamic conditions as mentioned above. At the end of 7 and 14 days, total RNA of cell/scaffolds samples were extracted by using RNeasy Mini Kit (QIAGEN, Hilden, Germany). The concentration of RNA was measured through NanoDrop™ One/OneC Microvolume UV–Vis Spectrophotometer (Thermofisher Scientific). mRNA of all samples was then transcribed into cDNA as templates in real-time PCR by iScriptTM cDNA Synthesis Kit (Bio-Rad Laboratories, Hercules, CA). Quantitative real-time PCR was performed afterwards by using iQ™ SYBR^®^ Green Supermix Kit (Bio-Rad Laboratories) on an AriaMx Real-Time PCR System (Agilent Technologies, Santa Clara, CA), and followed the instructions according to the manufacturers. Specific primers (expressed by italic, lower cases) including runt-related transcription factor 2 (*Runx2*), alkaline phosphatase (*alp*), bone sialoprotein (*bsp*), osteocalcin (*oc*), and glyceraldehyde 3-phosphate dehydrogenase (*GAPDH*) with the sequences shown in (Table 1) were purchased from Invitrogen. The $2^{{ - \Delta \Delta C_{T} }}$ method was used to relatively quantify genes expression, where $\Delta \Delta C_{T} = \left( {C_{T, Multiple} - C_{T,Non} } \right)_{Target\;gene} - \left( {C_{T, Multiple} - C_{T,Non} } \right)_{GAPDH}$, in which expressed target genes were normalized to the expression level of a housekeeping gene GAPDH^45,46^.

**Table 1:** Sequences of primers used for real-time PCR analysis.

Genes	Sequences	
GAPDH	For:	5′-AAC AGC GAC ACC CAC TCC TC
	Rev:	5′-CATACCAGGAAATGAGCTTGACAA
*Runx-2*	For:	5′-AGATGATGACACTGCCACCTCTG
	Rev:	5′-GGGATGAAATGCTTGGGAACT
*alp*	For:	5′-ACATTCCCACGTCTTCACATTT
	Rev:	5′-AGACATTCTCTCGTTCACCGCC
*bsp*	For:	5′-ATGGCCTGTGCTTTCTCAATG
	Rev:	5′-GGATAAAAGTAGGCATGCTTG
*oc*	For:	5′-TGTGAGCTCAATCCGGACTGT
	Rev:	5′-CCGATAGGCCTCCTGAAGC

### Immunofluorescent staining

The samples were immuno-probed with FAK and YAP-1 antibodies by following the instruction of manufacturers to investigate whether the addition of multiple channels inside scaffolds initiates the markers along with mechanotransduction pathway of hBMSCs. Briefly, the immunofluorescent staining of FAK and YAP-1, both non-channeled and multiple-channeled scaffolds were incubated with primary anti-FAK (1:100) and anti-YAP-1 (1:50) antibodies (Abcam, Cambridge, UK) separately at 4 °C overnight, followed with a subsequently fluorescent conjugated secondary antibody Alexa Fluor 594 (Invitrogen) labeling for 2 h at room temperature. F-actin fiber of cells on the scaffolds was stained by a fluorescent Phalloidin kit (Cytoskeleton, Denver, CO). Briefly, Actin-stain 555 phalloidin solution was used for the incubation of two types of scaffolds at room temperature for 30 min by following the instruction of manufacturer. After that, all the samples were co-stained with 4′6-diamidino-2-phenylindole (DAPI). The stained scaffolds were then placed on a glass cover slide on an inverted fluorescent microscope. Stained cells on the scaffolds were observed and imaged through a Nikon TE-2000 fluorescent microscope. Three random regions of each type of scaffold from the bottom to an inner area of the scaffold that the lens reached were pictured. The number of positively stained cells by FAK, YAP-1, and F-actin, and the total cells of each image per type of scaffolds were semi-quantified by FIJI ImageJ (NIH). The ratio of positively stained cells to total cells were calculated.

### In vivo implantation and fluorochrome labeling

#### Animals and ethical aspect

This study was carried out in compliance with the ARRIVE (Animal Research: Reporting In Vivo Experiments) guidelines. All the experiments were performed in compliance with the guidance and regulations of the Institutional Animal Care and Use Committee (IACUC) of Florida Atlantic University (FAU). All the experimental protocols were approved by the ethical committee of FAU IACUC, and the approved IACUC protocol number is A16-30. Nine female and nine male Wistar rats with the body weight 226–250 g were purchased from Charles River laboratories (Wilmington, MA).

#### In vivo implantation

To evaluate the function of the channel-pore architecture on bone regeneration, scaffolds were implanted in rat critical-sized calvarial bone defects. The animals were randomly divided to three groups, including non-channeled, multiple channeled scaffolds, and no-implant as a control. Six rats were used for each group (three female and three male rats). A circular 8 mm defect in diameter was created on each rat’s calvarial bone using a dental drill, and a scaffold with 8 mm in diameter and 1.5 mm in height was implanted. All the scaffold samples were shaped with sandpapers, washed with 70% ethanal and 1 × PBS for 3 times each, and autoclaved before utilization in the implantation surgery.

Fluorochrome labeling was performed postoperatively. Alizarin red and Calcein green were injected subcutaneously at week 6 and 10 weeks separately after the surgery. Both fluorochromes were purchased from Sigma-Aldrich (Munich, Germany), and solutions was prepared as shown in (Table 2). Before injections, both fluorochrome solutions were adjusted to pH 7.4 and sterilized through a 0.22 µm filter. The dosage was calculated in accordance with the body weight (BW) of each rat.

**Table 2:** Injection schedule and dosage of polyfluorochrome markers.

Time (weeks)	Fluorochrome	Dosage SC (mg/kg BW)	Solvent
6	Alizarin red	30	PBS/DI water (50/50)(v/v)
10	Calcein green	15	1 M NaOH

### Tissue harvest, fixation and X-ray characterization

All the animals were euthanized with 5% CO_2_ after 3 months of surgery. The entire defect with implanted scaffolds, and adjacent native bone tissue were harvested, rinsed with 1 × PBS, and fixed with 10% buffered formalin solution (Thermofisher Scientific) for 48 h. All the samples were characterized by a SkyScan microCT machine (Bruker, Billerica, MA) associated with the digital image analysis software (CT Analyser Version 1.18.4.0, SkyScan, Bruker microCT) to evaluate the regeneration and osseointegration of new bone on different implants.

### Histological and histomorphometric analysis

All harvested samples were processed for histological analysis by decalcifying with a rapid decalcifier solution (RDO Rapid Decalcifier, IL) for 7 days until the samples were softened. The samples were then embedded with paraffin and sectioned laterally and transversely. Hematoxylin and eosin (H&E) staining was carried out to evaluate newly formed bone density and height. For newly formed bone density analysis, each implant was analyzed from proximal, middle, and distal three transversal segments, and 0.5 mm height for each segment (bottom, middle, top). Three different regions of each segmental section were calculated, and three samples of each type of implant was considered. For all the other semi-quantitative evaluations, only central lateral segmental sections were considered for each implant.

### Immunohistochemical characterization

The central lateral sections of each group sample were probed with collagen type I, bone sialoprotein (BSP), osteocalcin (OC), and angiogenic marker CD31. Briefly, the samples were retrieved with 1 × citrate buffer (Sigma-Aldrich, St. Louis, MO) for 30 min at 95–100 °C. Followed which, all sets of groups were incubated with primary anti-BSP (1:500), anti-collagen type I (1:250), anti-OC (1:100), and anti-CD31 (1:50) antibodies (Abcam, Cambridge, UK) separately in a humidity chamber at 4 °C for overnight, and were subsequently marked with anti-biotinylated binding IgG secondary antibodies for at room temperature for 30 min. All immunohistochemical images were taken from a Nikon TE-2000 microscope, and three regions of each section were captured for semi-quantitative analysis using FIJI ImageJ (NIH). For BSP, collagen type I, and OC staining, percentage of positively stained tissue area to the whole tissue area were utilized on osteogenesis analysis. For CD 31 staining, the amount of blood vessels that formed on tissues were calculated for angiogenesis analysis^47,48^. All the other chemicals which were used in the characterizations were purchased from Vector Laboratories (CA, US).

### Statistical analysis

GraphPad Prism 6 (GraphPad, San Diego, CA) was used for conducting statistical analysis and plotting graphs. ANOVA with Tukey multiple comparison test was applied, and the results between groups are statistically significant when *p* < 0.05. All the experiments were performed in triplicates using one hBMSC donor.

## References

[1] Suenaga H, Furukawa KS, Suzuki Y, Takato T, Ushida T (2015). Bone regeneration in calvarial defects in a rat model by implantation of human bone marrow-derived mesenchymal stromal cell spheroids. J. Mater. Sci. Mater. Med..

[2] Zhang D (2017). Engineering biomimetic periosteum with beta-TCP scaffolds to promote bone formation in calvarial defects of rats. Stem Cell Res. Ther..

[3] Adamzyk C (2016). Bone tissue engineering using polyetherketoneketone scaffolds combined with autologous mesenchymal stem cells in a sheep calvarial defect model. J. Craniomaxillofac. Surg..

[4] Shin, S. Y., Rios, H. F., Giannobile, W. V. & Oh, T.-J. Periodontal regeneration: Current therapies. *Stem Cell Biology and Tissue Engineering in Dental Sciences* (edited by Vishwakarma, A, Sharpe, P., Shi, S. & Ramalingam, M.)(Academic Press). 459–469 (2015).

[5] Oryan A, Alidadi S, Moshiri A, Maffulli N (2014). Bone regenerative medicine: Classic options, novel strategies, and future directions. J. Orthop. Surg. Res..

[6] Mahyudin F (2017). Comparative effectiveness of bone grafting using xenograft freeze-dried cortical bovine, allograft freeze-dried cortical New Zealand white rabbit, xenograft hydroxyapatite bovine, and xenograft demineralized bone matrix bovine in bone defect of femoral diaphysis of white rabbit: Experimental study in vivo. Int. J. Biomater..

[7] Sheikh Z (2017). Natural graft tissues and synthetic biomaterials for periodontal and alveolar bone reconstructive applications: A review. Biomater. Res..

[8] Podaropoulos L, Veis AA, Papadimitriou S, Alexandridis C, Kalyvas D (2009). Bone regeneration using beta-tricalcium phosphate in a calcium sulfate matrix. J. Oral Implantol..

[9] Sohn HS, Oh JK (2019). Review of bone graft and bone substitutes with an emphasis on fracture surgeries. Biomater. Res..

[10] Bastami F (2017). Fabrication of a three-dimensional beta-tricalcium-phosphate/gelatin containing chitosan-based nanoparticles for sustained release of bone morphogenetic protein-2: Implication for bone tissue engineering. Mater. Sci. Eng. C Mater. Biol. Appl..

[11] Novosel EC, Kleinhans C, Kluger PJ (2011). Vascularization is the key challenge in tissue engineering. Adv. Drug Deliv. Rev..

[12] Laschke MW, Menger MD (2012). Vascularization in tissue engineering: Angiogenesis versus inosculation. Eur. Surg. Res..

[13] Takebe T (2012). Generation of functional human vascular network. Transplant. Proc..

[14] Zhao X (2016). In vitro vascularization of a combined system based on a 3D printing technique. J. Tissue Eng. Regen. Med..

[15] Nishiguchi A, Matsusaki M, Asano Y, Shimoda H, Akashi M (2014). Effects of angiogenic factors and 3D-microenvironments on vascularization within sandwich cultures. Biomaterials.

[16] Yu T (2016). Channeled β-TCP scaffolds promoted vascularization and bone augmentation in mandible of beagle dogs. Adv. Funct. Mater..

[17] Wang X, Lin M, Kang Y (2019). Engineering porous beta-tricalcium phosphate (beta-TCP) scaffolds with multiple channels to promote cell migration, proliferation, and angiogenesis. ACS Appl. Mater Interfaces.

[18] Liu YS (2015). Mechanosensitive TRPM7 mediates shear stress and modulates osteogenic differentiation of mesenchymal stromal cells through Osterix pathway. Sci. Rep..

[19] Wittkowske C, Reilly GC, Lacroix D, Perrault CM (2016). In vitro bone cell models: Impact of fluid shear stress on bone formation. Front. Bioeng. Biotechnol..

[20] Li Z (2013). Differential regulation of stiffness, topography, and dimension of substrates in rat mesenchymal stem cells. Biomaterials.

[21] Lee EA, Im SG, Hwang NS (2014). Efficient myogenic commitment of human mesenchymal stem cells on biomimetic materials replicating myoblast topography. Biotechnol. J..

[22] Liu Y (2010). Novel template-casting technique for fabricating beta-tricalcium phosphate scaffolds with high interconnectivity and mechanical strength and in vitro cell responses. J. Biomed. Mater. Res. A.

[23] Freed LE (1994). Biodegradable polymer scaffolds for tissue engineering. Biotechnology (N. Y).

[24] Chocholata P, Kulda V, Babuska V (2019). Fabrication of scaffolds for bone-tissue regeneration. Materials (Basel).

[25] Wang W, Yeung KWK (2017). Bone grafts and biomaterials substitutes for bone defect repair: A review. Bioact. Mater..

[26] Mansour A, Mezour MA, Badran Z, Tamimi F (2017). (*) Extracellular matrices for bone regeneration: A literature review. Tissue Eng. Part A.

[27] De Witte TM, Fratila-Apachitei LE, Zadpoor AA, Peppas NA (2018). Bone tissue engineering via growth factor delivery: From scaffolds to complex matrices. Regen. Biomater..

[28] Khojasteh A (2016). Development of PLGA-coated β-TCP scaffolds containing VEGF for bone tissue engineering. Mater. Sci. Eng. C.

[29] Kapur S, Baylink DJ, Lau KH (2003). Fluid flow shear stress stimulates human osteoblast proliferation and differentiation through multiple interacting and competing signal transduction pathways. Bone.

[30] Nishimura R, Hata K, Matsubara T, Wakabayashi M, Yoneda T (2012). Regulation of bone and cartilage development by network between BMP signalling and transcription factors. J. Biochem..

[31] Komori T (1997). Targeted disruption of Cbfa1 results in a complete lack of bone formation owing to maturational arrest of osteoblasts. Cell.

[32] Bjerre L, Bunger CE, Kassem M, Mygind T (2008). Flow perfusion culture of human mesenchymal stem cells on silicate-substituted tricalcium phosphate scaffolds. Biomaterials.

[33] Yourek G, McCormick SM, Mao JJ, Reilly GC (2010). Shear stress induces osteogenic differentiation of human mesenchymal stem cells. Regen. Med..

[34] Kreke MR, Sharp LA, Lee YW, Goldstein AS (2008). Effect of intermittent shear stress on mechanotransductive signaling and osteoblastic differentiation of bone marrow stromal cells. Tissue Eng. Part A.

[35] Sart S, Agathos SN, Li Y, Ma T (2016). Regulation of mesenchymal stem cell 3D microenvironment: From macro to microfluidic bioreactors. Biotechnol. J..

[36] Polo-Corrales L, Latorre-Esteves M, Ramirez-Vick JE (2014). Scaffold design for bone regeneration. J. Nanosci. Nanotechnol..

[37] Roach HI (1994). Why does bone matrix contain non-collagenous proteins? The possible roles of osteocalcin, osteonectin, osteopontin and bone sialoprotein in bone mineralisation and resorption. Cell Biol. Int..

[38] Davies E (2014). Citrate bridges between mineral platelets in bone. Proc. Natl. Acad. Sci. U. S. A..

[39] Fisher LW, Torchia DA, Fohr B, Young MF, Fedarko NS (2001). Flexible structures of SIBLING proteins, bone sialoprotein, and osteopontin. Biochem. Biophys. Res. Commun..

[40] Ganss B, Kim RH, Sodek J (1999). Bone sialoprotein. Crit. Rev. Oral Biol. Med..

[41] Bernards MT, Qin C, Jiang S (2008). MC3T3-E1 cell adhesion to hydroxyapatite with adsorbed bone sialoprotein, bone osteopontin, and bovine serum albumin. Colloids Surf. B Biointerfaces.

[42] Gordon JA, Hunter GK, Goldberg HA (2009). Activation of the mitogen-activated protein kinase pathway by bone sialoprotein regulates osteoblast differentiation. Cells Tissues Organs.

[43] Kang Y, Kim S, Bishop J, Khademhosseini A, Yang Y (2012). The osteogenic differentiation of human bone marrow MSCs on HUVEC-derived ECM and beta-TCP scaffold. Biomaterials.

[44] Kang Y, Kim S, Khademhosseini A, Yang Y (2011). Creation of bony microenvironment with CaP and cell-derived ECM to enhance human bone-marrow MSC behavior and delivery of BMP-2. Biomaterials.

[45] Kang Y, Kim S, Fahrenholtz M, Khademhosseini A, Yang Y (2013). Osteogenic and angiogenic potentials of monocultured and co-cultured human-bone-marrow-derived mesenchymal stem cells and human-umbilical-vein endothelial cells on three-dimensional porous beta-tricalcium phosphate scaffold. Acta Biomater..

[46] Livak KJ, Schmittgen TD (2001). Analysis of relative gene expression data using real-time quantitative PCR and the 2(-Delta Delta C(T)) method. Methods.

[47] Kang Y, Mochizuki N, Khademhosseini A, Fukuda J, Yang Y (2015). Engineering a vascularized collagen-beta-tricalcium phosphate graft using an electrochemical approach. Acta Biomater..

[48] Kang Y, Ren L, Yang Y (2014). Engineering vascularized bone grafts by integrating a biomimetic periosteum and beta-TCP scaffold. ACS Appl. Mater. Interfaces.

